# β-Lactam Inoculum Effect in Methicillin-Susceptible *Staphylococcus aureus* Infective Endocarditis

**DOI:** 10.1001/jamanetworkopen.2024.51353

**Published:** 2024-12-20

**Authors:** Baptiste Jean, Maelys Crolle, Candice Pollani, Adèle Le Guilloux, Guillaume Martin-Blondel, Pierre Tattevin, Audrey Le Bot, David Luque Paz, François Guérin, Vincent Cattoir, Laurence Armand-Lefevre, Signara Gueye, François-Xavier Lescure, Xavier Duval, Clémence Massip, Pierre Delobel

**Affiliations:** 1 Service des Maladies Infectieuses et Tropicales, Centre Hospitalier Universitaire (CHU) de Toulouse, Université Paul Sabatier Toulouse III, Toulouse, France; 2 Laboratoire de Bactériologie-Hygiène, CHU de Toulouse, Université Paul Sabatier Toulouse III, Toulouse, France; 3 Unité Méthodologie, Data Management, Analyses Statistiques, Centre d’Investigation Clinique 1436, Service de pharmacologie médicale, CHU de Toulouse, Toulouse, France; 4 Institut Toulousain des Maladies Infectieuses et Inflammatoires (Infinity), Institut National de la Santé et de la Recherche Médicale (INSERM) UMR 1291, Centre National de la Recherche Scientifique UMR 5051, Université Toulouse III, Toulouse, France; 5 Service des Maladies Infectieuses et Réanimation Médicale, CHU de Rennes, Université de Rennes, Rennes, France; 6 Service des Maladies Infectieuses et Réanimation Médicale, CHU de Rennes, Rennes, France; 7 Service de Bactériologie et Hygiène Hospitalière, CHU Rennes, Rennes, France; 8 Laboratoire de Bactériologie, Hôpital Bichat-Claude Bernard, Assistance Publique-Hôpitaux de Paris (AP-HP), Université Paris Cité, Infection, Anti-Microbien, Modélisation, Evolution (IAME), INSERM UMR 1137, Paris, France; 9 Service des Maladies Infectieuses et Tropicales, CHU Bichat, Université Paris Cité, IAME, INSERM UMR 1137, Paris, France; 10 Centre d’Investigation Clinique, AP-HP, Hôpital Bichat, INSERM Centre d'Investigation Clinique 1425, Université Paris Cité, IAME, INSERM, Paris, France

## Abstract

**Importance:**

Infective endocarditis (IE) caused by *Staphylococcus aureus* is associated with high mortality, approximately 20% to 30%, mostly in the first month, with no improvement in recent decades. Current opinion is that antistaphylococcal penicillin and cefazolin are equally effective in treating methicillin-susceptible *S aureus* (MSSA) IE, and both are recommended as possible first-line treatments. Most MSSA strains carry the β-lactamase *blaZ* gene, and some *blaZ*-positive strains exhibit an inoculum effect, meaning increased minimum inhibitory concentrations at high inoculum. This reduced susceptibility to an antibiotic at high bacterial inoculum may be particularly relevant in IE, where vegetations have very high bacterial densities.

**Objective:**

To evaluate the association between phenotypic characteristics of *S aureus* isolates, β-lactam used, and outcome in patients with MSSA IE.

**Design, Settings, and Participants:**

This retrospective case series included MSSA cases treated at 3 French university hospitals between February 2016 and February 2022. The study included patients who had clinical isolates available and had definite or possible *S aureus* IE that involved native or prosthetic valves. Data were analyzed from July 2023 to June 2024.

**Main Outcomes and Measures:**

MSSA isolates were tested for the presence of *blaZ* and for inoculum effects to cefazolin and oxacillin. The association between first-month mortality and the β-lactam used, the presence of *blaZ*, and the presence of an inoculum effect to the treatment received was evaluated.

**Results:**

This study included 216 patients with MSSA IE (median [IQR] age, 65 [49-73] years; 152 [70.4%] male) who were treated with antistaphylococcal penicillin (139 [64.4%]) or cefazolin (77 [35.6%]). One-month mortality of left-sided IE was 44 of 180 patients (24.4%), with no overall difference between patients treated with antistaphylococcal penicillin or cefazolin. However, 1-month mortality was higher in patients infected with *blaZ*-positive strains than with *blaZ*-negative strains (38 of 129 [29.5%] vs 6 of 51 [11.8%]; *P* = .01), and with strains with an inoculum effect to the β-lactam received than with strains without an inoculum effect (25 of 62 [40.3%] vs 13 of 67 [19.4%]; *P* = .005). On multivariable analysis, the presence of an inoculum effect was independently associated with first-month mortality (HR, 2.84; 95% CI, 1.28-6.30; *P* = .01).

**Conclusions and Relevance:**

In this case series of MSSA IE, the presence of an inoculum effect to the β-lactam received was a risk factor for death in the first month. Phenotyping MSSA isolates for inoculum effect may guide β-lactam choice and improve outcomes.

## Key Points

**Question:**

Is the clinical outcome of methicillin-susceptible *Staphylococcus aureus *(MSSA) infective endocarditis associated with some underappreciated phenotypic characteristics of the bacterial strains and the β-lactam used?

**Findings:**

In this case series of 216 patients with MSSA, first-month mortality in infective left-sided endocarditis due to MSSA strains appeared to be independently associated with the presence of *blaZ* and an inoculum effect, and this may account for some differences in outcomes between antistaphylococcal penicillin and cefazolin.

**Meaning:**

These findings suggest that characterizing the presence of *blaZ* and an inoculum effect may help to select the best treatment for MSSA infective endocarditis and improve clinical outcomes.

## Teaser

This case series evaluates the association between phenotypic characteristics of *Staphylococcus aureus* isolates, β-lactam used, and outcome in patients with methicillin-susceptible *S aureus* (MSSA) infective endocarditis at 3 hospitals in France.

## Introduction

Infective endocarditis (IE) caused by *Staphylococcus aureus* is associated with high mortality, estimated at 20% to 30% within 6 months. This mortality rate has remained stable since the 1980s.^1^ In Europe, methicillin resistance has decreased significantly over the past 2 decades, and most IEs are now caused by methicillin-susceptible *S aureus* (MSSA). The 2023 European Society of Cardiology recommendations suggest cefazolin or antistaphylococcal penicillin (ASP) as first-line treatment.^2^

Most clinical *S aureus* isolates carry the *blaZ* gene encoding a class-A β-lactamase (penicillinase). Four types of BlaZ β-lactamases (from A to D) have been characterized on the basis of substrate specificity and amino acid sequence.^3,4^ Some *blaZ*-positive *S aureus* strains exhibit an inoculum effect (ie, increased minimum inhibitory concentrations [MICs] at high inoculum), which may be clinically relevant in IE because of the high inoculum in vegetations.^5^ Despite the description of an inoculum effect to cefazolin, the overall outcomes of cefazolin have been found to be similar to that of ASP in several cohorts of MSSA IE.^6,7,8^ More recently, an inoculum effect to oxacillin has also been described, but its clinical impact remains unknown.^9^ The aims of this study are to investigate the characteristics of MSSA isolates responsible for IE, in particular the presence of *blaZ*, cefazolin inoculum effect, and oxacillin inoculum effect, and to analyze the association between these characteristics and the occurrence of death within 1 month of follow-up.

## Methods

### Study Design

This retrospective, multicenter case series included patients with MSSA IE between February 2016 and February 2022 at 3 French university hospitals located in 3 different regions, each with a cardiac surgery department (Paris-Bichat; Rennes; Toulouse). Patients were identified by cross-referencing data from the Programme de Médicalisation des Systèmes d’Information, coded as endocarditis, with the list of blood cultures positive for *S aureus*. For 1 center, patients were identified from a previously established cohort. This case series follows the Strengthening the Reporting of Observational Studies in Epidemiology (STROBE) reporting guideline.

#### Ethics Statement

The project has been registered with the French National Commission for Information and Freedom (Commission Nationale de l’Information et des Libertés) and approved by the French Committee on Ethics in Research on Infectious and Tropical Diseases (Comité d’Ethique de Recherche en Maladies Infectieuses et Tropicales No. 2023 0703). In accordance with French regulations for retrospective noninterventional studies, living participants were invited by letter to express any refusal to have their medical data included in the study. All data were anonymized.

#### Inclusion and Exclusion Criteria

Only patients with definite or possible *S aureus* IE according to the Duke 2015 criteria, involving native or prosthetic valves and for whom clinical isolates were available were included. Exclusion criteria for the analysis were IE on cardiac implantable electronic devices (CIED), vascular prosthesis infection, polymicrobial IE, methicillin-resistant *S aureus* (MRSA) IE, and patients who either did not receive β-lactam, or were treated for less than 72 hours with any of the β-lactam agents of interest (ASP and cefazolin) or whose targeted treatment with ASP or cefazolin was initiated after 120 hours.

### Data Collection

Data were collected from electronic medical records using a standardized questionnaire, and bacteriologic data were extracted from each center’s electronic laboratory record. Some variables used to calculate the Sequential Organ Failure Assessment (SOFA) score were scored as 0 in the absence of data in the patient’s electronic medical record (eg, absence of ratio of arterial oxygen partial pressure to the fraction of inspired oxygen [ie, PaO_2_/FiO_2_] data because arterial gasometry was not performed). The SOFA score was not calculated for patients who were transferred to 1 of the recruiting centers more than 72 hours after initiation of the β-lactam of interest.

### Bacteriological Characterization of *S aureus* Strains

All bacterial strains were centralized in the laboratory of the University Hospital of Toulouse for determination of *blaZ* type and inoculum effect testing. Bacterial DNA from each strain was extracted from colonies obtained after overnight incubation on Columbia blood agar at 35 °C ± 2 °C. DNA extraction was performed using enzymatic (mutanolysin) and thermal lysis methods. As previously described,^10^ a 355-bp region of the *blaZ* gene was amplified and sequenced using the following primers: *CAAAGATGATATAGTTGCTTATTC* and *CATATGTTATTGCTTGCACCAC*. Sequence analysis was performed using SnapGene version 6.0 (SnapGene) software. The β-lactamase type was determined based on the amino acids at positions 128 and 216 encoded by the *blaZ* gene.^3^ *S aureus* ATCC 29213 (a low activity type A β-lactamase producer) and *S aureus* ATCC 25923 (a β-lactamase negative strain) were used as positive and negative controls, respectively. Phylogenetic analysis was performed by aligning the sequences of the polymerase chain reaction products using MAFFT version 7.505 (Research Institute for Microbial Diseases). The maximum likelihood tree was then constructed with IQ-TREE version 2.0.3,^11^ using the HKY+F evolutionary model (ModelFinder)^12^ and the ultrafast bootstrap (UFBoot2)^13^ for branch support.

Bacterial identification was confirmed by matrix-assisted laser desorption/ionization time-of-flight mass spectrometry. Cefazolin and oxacillin MICs were determined for *blaZ*-positive strains by the broth microdilution reference method according to European Committee on Antimicrobial Susceptibility Testing (EUCAST) recommendations, with the following exceptions: MICs were determined using standard (2.10^5^ CFU per ml) and high (2.10^7^ CFU per ml) inocula. As recommended, sodium chloride (2 g of solute per 100 mL of solution) was added in the cation-adjusted Muller Hinton broth for oxacillin testing. The *S aureus* ATCC 29213 reference strain was used as a control. All MICs were determined in duplicate and read by 2 observers blinded to the type of *blaZ*. If there was a single dilution discrepancy between the 2 independent MIC determinations, a triplicate was performed. Replicates never differed by more than 2-fold. Inoculum effect was defined as a 4-fold increase in MIC between standard and high inoculum.^14^

### Statistical Analysis

Descriptive statistics were presented using No. (%) for categorical variables and median (IQRs) for quantitative variables. The χ^2^ test was used to compare the distribution of the genotypic characteristics of *blaZ* (absence, A, B, C) between the 3 centers (Paris-Bichat; Rennes; Toulouse) and to test associations between *blaZ* (presence and type) and inoculum effect to cefazolin and ASP.

Survival analysis was performed with the primary end point being the occurrence of death in the first month: event was the death (all causes), and delay was the time between the start of antibiotic treatment (including probabilistic treatment before targeting to cefazolin or ASP) and the death or the censoring (day 30). Survival curves were plotted using the Kaplan-Meier method and compared by treatment using the log-rank test. The same methods were used to compare the survival curves between *blaZ* (presence and type) and the survival curves between inoculum effect to cefazolin and oxacillin.

Univariable and multivariable Cox regression models were constructed to identify factors associated with the occurrence of death in the first month after the start of antibiotic treatment. Potential factors were: Charlson comorbidity index (total quantitative score), age (years), history of stroke and transient ischemic attack (presence or absence), pulmonary pathology (presence or absence), diabetes (presence or absence), chronic kidney failure (presence or absence), SOFA score (total quantitative score), surgery within the first 15 days (yes or no), vegetation size (mm), perforation or regurgitation (yes or no); inoculum effect (presence of inoculum effect or absence of *blaZ* or inoculum effect). Each model (univariable and multivariable) was adjusted for β-lactam treatment received. First, each factor was tested in a univariable model. Second, a complete multivariable model was constructed. The variable surgery within the first 15 days, previously identified in the literature as a protective factor, was included in the multivariable model, although it was not significantly associated with mortality in the univariate analysis. Finally, factors with a *P* < .20 were included to obtain a final multivariable model using the stepwise selection method. The proportional hazards assumption was tested by plotting Schoenfeld residuals against event times for each of the factors in each model. Sensitivity analyses were also performed: 1 with adjustment for clinical center instead of β-lactam treatment and the other by replacing the Charlson comorbidity index (total quantitative score) with the variables included in it (ie, age, history of stroke and transient ischemic attack, pulmonary pathology, diabetes, chronic kidney failure). Because of the lower mortality in patients with right-sided IE (2.8% in right-sided vs 24.4% in left-sided IE), all survival analyses were performed only in the 180 patients with left-sided IE, and Cox regression models were restricted to the 122 patients with complete data for the variables analyzed, rather than imputing values for missing data.

The α-risk was set at 5% (*P* < .05). All tests were 2-sided. All analyses were performed using R version 4.3.1 (R Project for Statistical Computing). Data were analyzed from July 2023 to June 2024.

## Results

### Population Description

**Figure 1.**
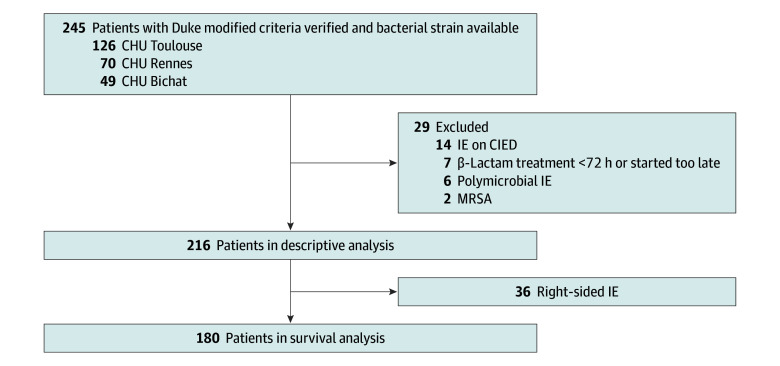
Study Flowchart CIED indicates cardiac implantable electronic devices; CHU, Centre Hospitalier Universitaire; IE, infective endocarditis.

We identified 245 patients with possible or definite MSSA IE who received treatment between February 2016 and February 2022 at the participating sites, for whom *S aureus* strains were still available in the bacteriology laboratory. Of these, 29 patients were subsequently excluded for the following reasons: IE on CIED (14 [50.0%]), polymicrobial IE (6 [21.4%]), final diagnosis of methicillin-resistant *S aureus* IE (2 [7.1%]), MSSA IE cases not receiving or receiving too late the β-lactam of interest (7 [25%]). A total of 216 patients were selected for the descriptive analysis (Figure 1).

The median (IQR) age of the patients was 65 (49-73) years; the male-to-female sex ratio was 2.4 (152 males [70.4%]; 64 females [29.6%]). Patients had a median (IQR) Charlson comorbidity index of 3 (1-6). Intravenous drug use was observed in 32 patients (14.8%). The median (IQR) SOFA score at baseline was 4 (2-7). According to the Duke 2015 criteria, 195 episodes (90.3%) met the definition of a definite IE, while 21 episodes (9.7%) met the definition of a possible IE. Most episodes of IE were due to aortic involvement (76 [35.2%]), followed by mitral (60 [27.8%]) and tricuspid (36 [16.6%]) involvement, while 27 episodes [12.5%] were polyvalvular IE. Additionally, 161 episodes (74.5%) occurred on native valves, and 55 episodes (25.5%) occurred on prosthetic valves. Vegetation size was reported in 166 cases (76.9%), with a median (IQR) size of 13 (8-20) mm. Mortality rates were 14.8% (32 of 216) at day 15 and 20.8% (45 of 216) at day 30. Regarding initial treatment, 77 patients (35.6%) received cefazolin, while 139 (64.4%) received ASP. The *blaZ* gene was carried by 159 strains (73.6%), and *blaZ* types A and B were equally represented, each accounting for 57 strains (26.4%), while type C accounted for 44 strains (20.4%). Only 1 strain (0.4%) carried *blaZ* type D (Table 1). There was no difference observed in the distribution of *blaZ* types between the 3 centers (eFigure 1 in Supplement 1).

**Table 1.** Description of Study Patients With MSSA Infective Endocarditis

Characteristic	Patients, No. (%) (N = 216)
Demographic data	
Age, median (IQR), y	65 (49-73)
Sex	
Male	152 (70.4)
Female	64 (29.6)
Intravenous drug user	32 (14.8)
Charlson comorbidity index, median (IQR)	3 (1-6)
SOFA score, median (IQR)	4 (2-7)
Unknown	16 (7.4)
Outcome data	
Neurological complications of infective endocarditis	86 (39.8)
Emboli or stroke	83 (38.4)
Hematoma or abscess	29 (13.4)
Mycotic aneurysm	7 (3.2)
Bacteremia duration, median (IQR), d	3.25 (1.2-5.9)
Unknown	2 (0.9)
Mortality rate	
Day 15	32 (14.8)
Day 30	45 (20.8)
Pathology data	
Modified Duke classification	
Definite endocarditis	195 (90.3)
Possible endocarditis	21 (9.7)
Affected valve	
Aortic	76 (35.2)
Mitral	60 (27.8)
Tricuspid	36 (16.6)
Multiple	27 (12.5)
Undetermined and other	17 (7.9)
Valve type	
Native valve	161 (74.5)
Prosthetic valve	55 (25.5)
Vegetation size, median (IQR), mm	13 (8-20)
Unknown	50 (23.1)
Intracardiac abscess	47 (21.8)
Perforation or regurgitation	34 (15.7)
Atrioventricular block grade III	19 (8.8)
Bacteriological data	
*blaZ* presence	159 (73.6)
*blaZ* type	
Absence	57 (26.4)
A	57 (26.4)
B	57 (26.4)
C	44 (20.4)
D	1 (0.4)
Cefazolin inoculum effect	
Absence	118 (54.6)
Presence	41 (19.0)
Not available^a^	57 (26.4)
Oxacillin inoculum effect	
Absence	77 (35.6)
Presence	82 (38.0)
Not available^a^	57 (26.4)
Treatment data	
B-lactam used	
Cefazolin	77 (35.6)
Antistaphylococcal penicillin	139 (64.4)
Surgery within the first 15 d	62 (28.7)
Aminoglycoside usage	73 (33.8)

MSSA, methicillin-susceptible *Staphylococcus aureus*; SOFA, Sequential Organ Failure Assessment.

^a^ *blaZ* Negative.

**Figure 2.**
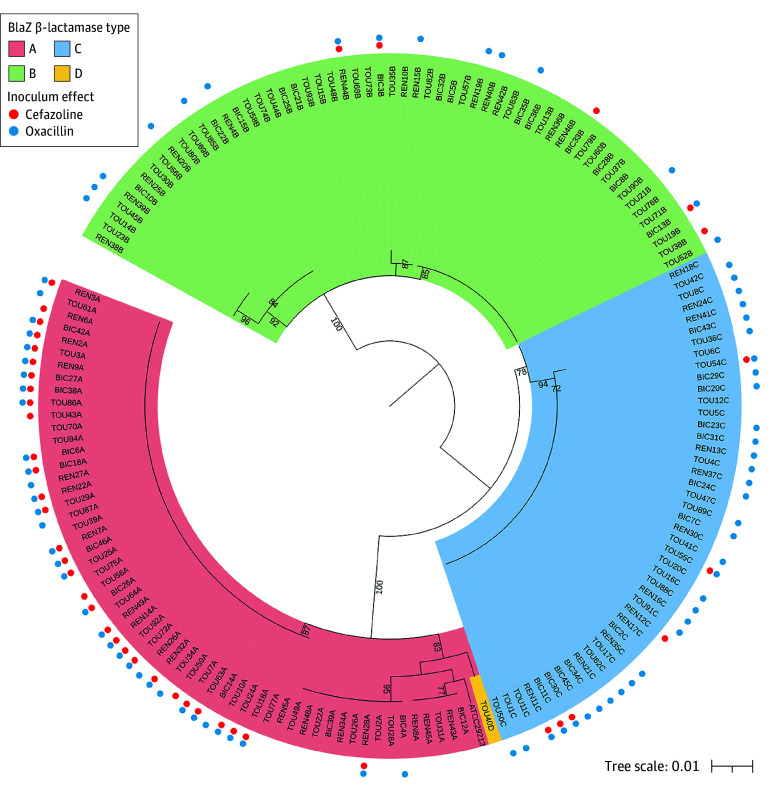
Phylogenetic Tree of the 159 *blaZ*-Positive *Staphylococcus aureus* Strains Maximum likelihood phylogenetic tree based on alignment of 355 bp polymerase chain reaction products from *blaZ* amplification. Strains with a cefazolin inoculum effect and strains with an oxacillin inoculum effect are marked with a red and blue dot, respectively. Bootstrap values are only shown above 70. The name of each strain corresponds to the first 3 letters of the hospital where it was isolated, followed by an increasing number for each hospital and finally the letter corresponding to the *blaZ* type. BIC indicates Bichat Hospital; REN, Rennes Hospital; TOU, Toulouse Hospital.

Oxacillin inoculum effect was observed in 82 of 159 *blaZ*-positive strains (51.6%), and 5 of 159 strains (3.1%) of strains had an oxacillin MIC greater than 2 mg/L at high inoculum. Cefazolin inoculum effect was observed in 41 of 159 *blaZ*-positive strains (25.8%). However, none of the strains were classified as resistant to cefazolin at high inoculum, ie, all the MICs were below the EUCAST breakpoint of 16 mg/L. Additionally of the 159 *blaZ*-positive strains, 34 (21.4%) had both oxacillin and cefazolin inoculum effect, 48 (30.2%) had OXA inoculum effect but not cefazolin inoculum effect, 7 (4.4%) had cefazolin inoculum effect but not oxacillin inoculum effect, and 70 (44.0%) had neither (*P* < .001). Oxacillin inoculum effect was associated with *blaZ* types A and C (33 of 57 *blaZ* type A strains [57.9%] and 34 of 44 *blaZ* type C strains [77.2%]), compared with only 15 of 57 *blaZ* type B strain (26.3%) (*P* < .001). Cefazolin inoculum effect was associated with *blaZ* type A (30 of 57 *blaZ* type A strains [52.6%]), compared with 5 of 57 *blaZ* type B strain (8.8%) and 6 of 44 *blaZ* type C strain (13.6%) (*P* < .001). Most *blaZ* type A strains with cefazolin inoculum effect had sequence similarities for the 355 bp examined (Figure 2).

### Phenotypic Characteristics of *blaZ*-Positive Strains and Treatment Outcome in Left-Sided IE

**Figure 3.**
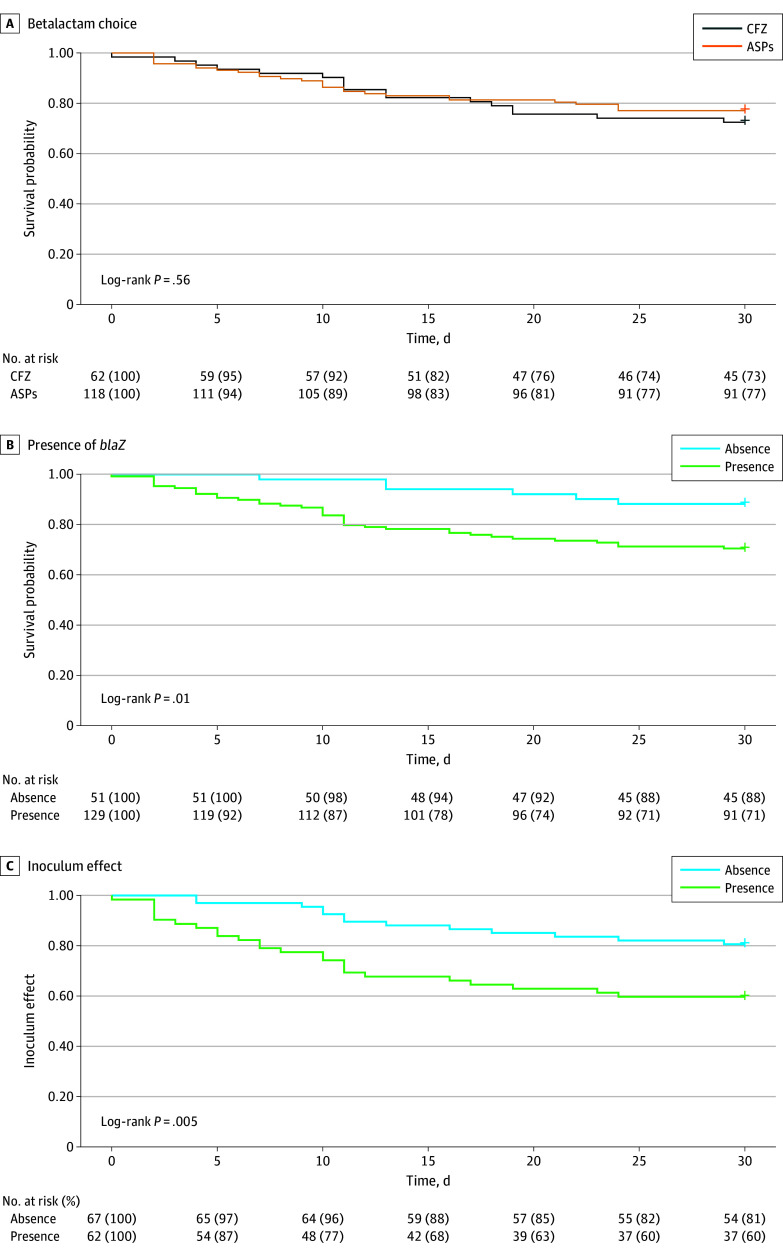
One-Month Survival Curves in Methicillin-Susceptible Staphylococcus aureus Left-Sided Infective Endocarditis Kaplan-Meier survival curves according to (A) the treatment with cefazolin (CFZ) or antistaphylococcal penicillin (ASP), (B) the presence or absence of *blaZ*, and (C) the presence or absence of an inoculum effect in the *blaZ*-positive group.

Because of the low mortality in the right-sided IE group (1 of 36 patients [2.8%]), our survival analyses focused on left-sided IE (mortality: 44 of 180 patients [24.4%]). The 1-month mortality in the cefazolin group and ASP group was not significant (17 of 62 patients [27.4%] vs 27 of 118 patients [22.9%], respectively; log-rank test *P* = .56) (Figure 3A). In contrast, 1-month mortality in the *blaZ*-positive group was 29.5% (38 of 129 patients) compared with 11.8% (6 of 51 patients) in the *blaZ*-negative group, regardless of which β-lactam they received (log-rank test *P* = .01) The difference in survival occurred mostly within the first 15 days of treatment, with a mortality rate of 5.9% (3 of 51 patients) in the *blaZ*-negative group vs 21.7% (28 of 129 patients) in the *blaZ*-positive group, reflecting a difference of 15.8% at day 15. At day 30, the mortality rate was 11.8% (5 of 51 patients) in the *blaZ*-negative group vs 29.5% (38 of 129 patients) in the *blaZ*-positive group, reflecting a difference of 17.7% at day 30 (Figure 3B). Among patients infected with *blaZ*-positive strains, there was no significant difference in the occurrence of death in the first month according to *blaZ* type (eFigure 2 in Supplement 1).

We then examined whether the presence of an inoculum effect to the treatment received was associated with the occurrence of death in the first month in the *blaZ*-positive group. One-month mortality was 25 of 62 patients (40.3%) in the group with inoculum effect–positive strains to the β-lactam received compared with 13 of 67 patients (19.4%) in the group with inoculum effect–negative strains (log-rank test *P* = .005) (Figure 3C). Mortality in the inoculum effect–negative group was similar to that in the *blaZ*-negative group (13 of 67 [19.4%] vs 6 of 51 [11.8%]; log-rank test *P* = .25).

The occurrence of death in the first month was also examined according to cefazolin and ASP subgroups. Among patients infected with a *blaZ*-positive strain and treated with cefazolin, there was a significant difference in 1-month mortality between patients infected with cefazolin inoculum effect–positive strains vs cefazolin inoculum effect–negative strains (8 of 15 [53.3%] vs 6 of 30 [20.0%]; log-rank test *P* = .01) (eFigure 3A in Supplement 1). Similarly, among patients infected with a *blaZ*-positive strain and treated with ASPs, there was a numerically greater 1-month mortality in patients infected with oxacillin inoculum effect–positive strains vs oxacillin inoculum effect–negative strains (17 of 47 [36.2%] vs 7 of 37 [18.9%]; log-rank test *P* = .06), but the difference was not statistically significant (eFigure 3B in Supplement 1). This outcome of inoculum effect on 1-month mortality was specific to the β-lactam used, as differences in 1-month mortality between the oxacillin inoculum effect–positive strain group and the oxacillin inoculum effect–negative strain group in cefazolin-treated patients (8 of 20 [40.0%] vs 6 of 24 [24.0%]; log-rank test *P* = .19) (eFigure 3C in Supplement 1), and between the cefazolin inoculum effect–positive and cefazolin inoculum effect–negative strain groups in patients receiving ASP (4 of 20 [20.0%] vs 20 of 64 [31.3%]; log-rank test *P* = .37) were smaller (eFigure 3D in Supplement 1).

### Risk Factors for Mortality in Left-Sided MSSA IE

Univariable and multivariable analyses of parameters associated with the occurrence of death in the first month of left-sided IE were performed using Cox regression. Table 2 summarizes the variables for which the univariable analysis showed an association with 1-month mortality with *P* < .20. In the final model of multivariable analysis, 4 variables remained independently associated with the occurrence of death in the first month, after adjustment for the β-lactam received: the Charlson comorbidity index (HR, 1.22; 95% CI, 1.10-1.36; *P* < .001); the SOFA score (HR, 1.14; 95% CI, 1.05-1.23; *P* = .001); the vegetation size (HR, 1.04; 95% CI, 1.01-1.07; *P* = .02); and the presence of an inoculum effect to the treatment received (HR, 2.84; 95% CI, 1.28-6.30; *P* = .01) (Table 2). Results were globally similar when adjusted for clinical center (eTable 1 in Supplement 1). Sensitivity analyses of multivariable Cox models including the variables comprising the Charlson comorbidity index instead of the total score were also performed (eTable 2 in Supplement 1).

**Table 2.** Univariable and Multivariable Cox Regression Models of Factors Associated With the Occurrence of First-Month Death in 122 Patients With Left-Sided Infective Endocarditis After Adjustment for Treatment Received

Variables	Univariable analysis adjusted for treatment received		Complete model 1 adjusted for treatment received		Model 2 with selection adjusted for treatment received	
	HR (95% CI)	*P* value	HR (95% CI)	*P* value	HR (95% CI)	*P* value
Charlson comorbidity index, per 1-point increase	1.18 (1.07-1.30)	.001	1.19 (1.06-1.33)	.002	1.22 (1.10-1.36)	<.001
Age, per 1-y increase	1.03 (1.00-1.05)	.04	NA	NA	NA	NA
History of stroke and transient ischemic attack (reference category, no history of stroke and transient ischemic attack)	2.05 (0.70-6.00)	.19	NA	NA	NA	NA
Pulmonary pathology (reference category, no pulmonary pathology)	2.59 (0.99-6.77)	.05	NA	NA	NA	NA
Diabetes (reference category, no diabetes)	1.33 (0.82-2.14)	.25	NA	NA	NA	NA
Chronic kidney failure (reference category, no kidney chronic failure)	3.36 (1.65-6.84)	.001	NA	NA	NA	NA
SOFA score, per 1-point increase	1.15 (1.07-1.24)	<.001	1.14 (1.05-1.24)	.001	1.14 (1.05-1.23)	.001
Surgery within the first 15 d (reference category, no surgery)	0.81 (0.38-1.72)	.59	0.52 (0.20-1.31)	.17	NS	NS
Vegetation size, per 1-mm increase	1.06 (1.02-1.09)	.002	1.05 (1.01-1.09)	.01	1.04 (1.01-1.07)	.02
Perforation or regurgitation (reference category, no perforation or regurgitation)	2.17 (0.98-4.81)	.06	1.74 (0.76-4.01)	.19	NS	NS
Inoculum effect (reference category, absence of *blaZ* or inoculum effect)	2.59 (1.25-5.35)	.01	2.80 (1.26-6.36)	.01	2.84 (1.28-6.30)	.01

Abbreviations: HR, hazard ratio; NA, not analyzed in these models because the global Charlson comorbidity index was used instead; NS, not selected during stepwise selection; SOFA, Sequential Organ Failure Assessment.

## Discussion

The main finding of this multicenter study was the independent association between *blaZ* presence and inoculum effect and the mortality of MSSA in left-sided IE. Indeed, the presence of an inoculum effect to the β-lactam received was an independent risk factor for the occurrence of death in the first month. Overall survival did not differ between the use of ASP and cefazolin, which was consistent with the results of several studies comparing ASP with cefazolin in MSSA bacteremia.^15,16,17,18^ However, in all these studies, ASPs and cefazolin were compared as a whole without separating each β-lactam received according to the presence or absence of *blaZ* and inoculum effect.

Inoculum effect to β-lactam antibiotics has been described mainly for cefazolin,^10,19^ but is less known for ASPs, although it was common in our study (about half of *blaZ*-positive strains). To our knowledge, the association between oxacillin inoculum effect and ASP treatment failure in MSSA infection has not been previously described. inoculum effect was associated with mortality only in patients treated with the β-lactam, showing inoculum effect against that β-lactam. These data support the idea of a hydrolytic specificity of BlaZ. The reduced susceptibility to oxacillin at high inoculum was consistent with the concept of borderline oxacillin-resistant *S aureus* (BORSA), where resistance to ASPs in these strains is associated with the overexpression of *blaZ*.^20^ Furthermore, *blaZ* expression can be regulated by exposure to β-lactam antibiotics.^21^ Therefore, exploring the hydrolytic profile of BlaZ and the amount of enzymes produced by different strains may help to optimize antibacterial treatment.

Our results showed a significant proportion of *blaZ*-negative strains, accounting for about one-fourth of this French cohort, supporting the results of previous studies^22,23^ indicating an increase in the frequency of *blaZ*-negative strains in other European countries, China, and the US. The prevalence of *blaZ*-positive isolates increased sharply in the decades following the widespread use of penicillin. The resurgence of *blaZ*-negative MSSA raised questions about the determinants involved in this dynamic. Furthermore, the identification of *blaZ*-negative isolates may enable the use of penicillin G against these strains. Notably, these strains have significantly lower penicillin G MICs compared with ASP and cefazolin.^24^ In addition, penicillin G has the potential to mitigate the environmental impact of prolonged antibiotic therapy for IE.

The influence of phenotypic characteristics of BlaZ on therapeutic outcome suggests new therapeutic perspectives. The use of phenotypic characteristics of MSSA isolates, such as inoculum effect determination, may help in the future to select the optimal β-lactam agent. It may also prompt consideration of adding β-lactamase inhibitors, such as clavulanic acid, to conventional therapy against β-lactamase-producing strains. While gene sequencing remains the reference diagnostic for *blaZ* detection and typing, its application in daily routine remains challenging. Moreover, it does not seem sufficient to estimate inoculum effect. By contrast, determining the presence of an inoculum effect to cefazolin or oxacillin could be implemented in routine practice by adapting commercial broth microdilution methods with standard and high inocula.

### Limitations

Our study has several limitations. As a retrospective and observational study, this study is at risk of bias due to factors such as selection and information bias. The clinical severity of the patients may have influenced the choice of treatment. However, this bias is partially mitigated by the predominant use of cefazolin from 2016 to 2018, during the prolonged and complete ASP shortage, which reduces the risk of indication bias. Another important parameter in IE treatment is achieving optimal antibiotic concentration in this context of high inoculum infection. Therapeutic drug monitoring was not systematically performed in our study, which may have affected therapeutic outcomes. Despite the reasonable sample size, the number of participants in subgroup analyses remains limited, which may affect the generalizability of our findings. In addition, there is a potential risk of confounding by circulating local clones, although the inclusion of multiple centers mitigates this risk. We performed adjustment for either the β-lactam received or the clinical center in Cox regression analyses to account for these potential biases, and in all models, the presence of *blaZ* and inoculum effect remained a factor independently associated with the occurrence of death in the first month. However, it should be emphasized that our results were obtained in the context of high-inoculum infection and may not apply to *S aureus* isolates responsible for other sites of infection with lower inoculum than in IE.

## Conclusions

In conclusion, left-sided MSSA IEs remain associated with high mortality rates, of 24.4% at 1 month, mostly within the first 2 weeks, highlighting the importance of early optimization of treatment strategies. Our study highlights the association between MSSA isolate characteristics, particularly the presence of *blaZ* and inoculum effect, the β-lactam agent used, and the outcomes, suggesting that the choice of β-lactam agent could be guided by phenotypic characteristics of the isolates based on early and reliable microbiological testing. In addition, the 25% prevalence of *blaZ*-negative isolates may allow greater use of penicillin G. Our results highlight the potential value of phenotypic testing of MSSA isolates to allow tailored optimization of antibacterial treatment in IE to improve outcome and prompt further consideration of its implications for other high-inoculum infections.

## Notes

Supplement 1. **eTable 1. **Univariable and Multivariable Cox Regression Models of Factors Associated With the Occurrence of First-Month Death in Left-Sided IE After Adjustment for Clinical Center **eTable 2. **Sensitivity Analyses of Multivariable Cox Regression Models of Factors Associated With the Occurrence of First-Month Death in Left-Sided IE **eFigure 1. ***blaZ* Type Frequency by Clinical Center **eFigure 2. **One-Month Survival Curves in MSSA Left-Sided Infective Endocarditis According to *blaZ *Type **eFigure 3. **One-Month Survival Curves in MSSA Left-Sided Infective Endocarditis in Patients Infected With a *blaZ*-Positive Strain According to the Presence or Absence of an Inoculum Effect to the β-Lactam Received

Supplement 2.
Data Sharing Statement
