# Supplementary material for: β-Lactam Inoculum Effect in Methicillin-Susceptible Staphylococcus aureus Infective Endocarditis
Source: JAMA Netw Open. 2024 Dec 20;7(12):e2451353. doi: 10.1001/jamanetworkopen.2024.51353 (PMC11662251; doi:10.1001/jamanetworkopen.2024.51353)
Supplement: Supplement 1. — eTable 1. Univariable and Multivariable Cox Regression Models of Factors Associated With the Occurrence of First-Month Death in Left-Sided IE After Adjustment for Clinical Center eTable 2. Sensitivity Analyses of Multivariable Cox Regression Models of Factors Associated With the Occurrence of First-Month Death in Left-Sided IE eFigure 1. blaZ Type Frequency by Clinical Center eFigure 2. One-Month Survival Curves in MSSA Left-Sided Infective Endocarditis According to blaZ Type eFigure 3. One-Month Survival Curves in MSSA Left-Sided Infective Endocarditis in Patients Infected With a blaZ-Positive Strain According to the Presence or Absence of an Inoculum Effect to the β-Lactam Received [file jamanetwopen-e2451353-s001.pdf]

Supplemental Online Content

Jean B, Crolle M, Pollani C, et al. β-lactam inoculum effect in methicillin-susceptible *Staphylococcus aureus* infective endocarditis. *JAMA Netw Open*. 2024;7(12):e2451353. doi:10.1001/jamanetworkopen.2024.51353

eTable 1. Univariable and Multivariable Cox Regression Models of Factors Associated With the Occurrence of First-Month Death in Left-Sided IE After Adjustment for Clinical Center
eTable 2. Sensitivity Analyses of Multivariable Cox Regression Models of Factors Associated With the Occurrence of First-Month Death in Left-Sided IE
eFigure 1. *blaZ* Type Frequency by Clinical Center
eFigure 2. One-Month Survival Curves in MSSA Left-Sided Infective Endocarditis According to *blaZ* Type
eFigure 3. One-Month Survival Curves in MSSA Left-Sided Infective Endocarditis in Patients Infected With a *blaZ*-Positive Strain According to the Presence or Absence of an Inoculum Effect to the β-Lactam Received

This supplemental material has been provided by the authors to give readers additional information about their work.

© 2024 Jean B et al. *JAMA Network Open.*

eTable 1: Univariable and multivariable Cox regression models of factors associated with the occurrence of first-month death in left-sided IE after adjustment for clinical center

N = 122	Univariable analysis adjusted for clinical center			Complete model 1 adjusted for clinical center			Model 2 with selection adjusted for clinical center		
**Variables**	**HR**	**[95% CI]**	**p value**	**HR**	**[95% CI]**	**p value**	**HR**	**[95% CI]**	**p value**
**Charlson comorbidity index**<br>(per one point increase)	1.17	[1.06-1.29]	**0.001**	1.17	[1.05-1.31]	**0.003**	1.21	[1.09-1.34]	**<0.001**
**Age**<br>(per one year increase)	1.03	[1.00-1.05]	0.05	NA					
**History of stroke and transient ischemic attack**<br>(reference=no)	2.10	[0.73-6.05]	0.17	NA					
**Pulmonary pathology**<br>(reference=no)	2.56	[0.98-6.71]	0.06	NA					
**Diabetes**<br>(reference=no)	1.32	[0.82-2.14]	0.25	NA					
**Chronic renal failure**<br>(reference=no)	3.34	[1.63-6.82]	**0.001**	NA					
**SOFA score**<br>(per one point increase)	1.13	[1.05-1.22]	**0.001**	1.12	[1.03-1.22]	**0.01**	1.12	[1.03-1.22]	**0.01**
**Surgery within the first 15 days**<br>(reference=no)	0.78	[0.37-1.68]	0.53	0.44	[0.16-1.21]	0.11	NS		
**Vegetation size**<br>(per one mm increase)	1.05	[1.02-1.09]	**0.003**	1.05	[1.01-1.09]	**0.02**	1.03	[1.00-1.07]	0.06
**Perforation or regurgitation**<br>(reference=no)	2.21	[1.00-4.87]	**0.05**	1.83	[0.78-4.28]	0.16	NS		
**Inoculum effect (InE)**<br>(reference= absence of *blaZ* or InE)	2.32	[1.14- 4.70]	**0.02**	2.26	[1.04-4.89]	**0.04**	2.25	[1.06-4.78]	**0.03**

NA: Not analyzed in the models because the global Charlson comorbidity index was used instead
NS: Not selected during stepwise selection

© 2024 Jean B et al. *JAMA Network Open.*

eTable 2. Sensitivity analyses of multivariable Cox regression models of factors associated with the occurrence of first-month death in left-sided IE

N = 122	Univariable analysis adjusted for treatment received			Complete model 1 adjusted for treatment received			Model 2 with selection adjusted for treatment received		
**Variables**	**HR**	**[95% CI]**	**p value**	**HR**	**[95% CI]**	**p value**	**HR**	**[95% CI]**	**p value**
**Charlson comorbidity index** (per one point increase)	1.18	[1.07-1.30]	**0.001**	NA					
**Age** (per one year increase)	1.03	[1.00-1.05]	**0.04**	1.03	[1.00-1.06]	**0.05**	1.03	[1.00-1.06]	0.06
**History of stroke and transient ischemic attack** (reference=no)	2.05	[0.70-6.00]	0.19	2.58	[0.76-8.79]	0.13	2.64	[0.87-8.05]	0.09
**Pulmonary pathology** (reference=no)	2.59	[0.99-6.77]	0.05	1.26	[0.44-3.59]	0.66	NS		
**Diabetes** (reference=no)	1.33	[0.82-2.14]	0.25	1.34	[0.76-2.36]	0.31	NS		
**Chronic renal failure** (reference=no)	3.36	[1.65-6.84]	**0.001**	0.85	[0.32-2.30]	0.75	NS		
**SOFA score** (per one point increase)	1.15	[1.07-1.24]	**<0.001**	1.14	[1.05-1.24]	**0.001**	1.14	[1.05-1.24]	**0.002**
**Surgery within the first 15 days** (reference=no)	0.81	[0.38-1.72]	0.59	0.52	[0.20-1.31]	0.17	0.39	[0.15-1.06]	0.07
**Vegetation size** (per one mm increase)	1.06	[1.02-1.09]	**0.002**	1.05	[1.01-1.09]	**0.01**	1.06	[1.02-1.10]	**0.003**
**Perforation or regurgitation** (reference=no)	2.17	[0.98-4.81]	0.06	1.74	[0.76-4.01]	0.19	2.07	[0.90-4.76]	0.09
**Inoculum effect (InE)** (reference= absence of *blaZ* or InE)	2.59	[1.25-5.35]	**0.01**	2.80	[1.26-6.36]	**0.01**	2.84	[1.28-6.30]	**0.01**

NA: Not analyzed in the models because the variables comprising the Charlson comorbidity index were used instead of the total score
NS: Not selected during stepwise selection

© 2024 Jean B et al. *JAMA Network Open.*

N = 122	Univariable analysis adjusted for clinical center			Complete model 1 adjusted for clinical center			Model 2 with selection adjusted for clinical center		
**Variables**	**HR**	**[95% CI]**	**p value**	**HR**	**[95% CI]**	**p value**	**HR**	**[95% CI]**	**p value**
**Charlson comorbidity index** (per one point increase)	1.17	[1.06-1.29]	**0.001**	NA					
**Age** (per one year increase)	1.03	[1.00-1.05]	0.05	1.03	[1.00-1.06]	0.07	1.03	[1.00-1.06]	0.07
**History of stroke and transient ischemic attack** (reference=no)	2.10	[0.73-6.05]	0.17	3.07	[0.88-10.69]	0.08	3.03	[1.00-9.17]	**0.05**
**Pulmonary pathology** (reference=no)	2.56	[0.98-6.71]	0.06	1.11	[0.38-3.26]	0.85	NS		
**Diabetes** (reference=no)	1.32	[0.82-2.14]	0.25	1.40	[0.81-2.43]	0.23	NS		
**Chronic renal failure** (reference=no)	3.34	[1.63-6.82]	**0.001**	0.87	[0.32-2.41]	0.79	NS		
**SOFA score** (per one point increase)	1.13	[1.05-1.22]	**0.001**	1.12	[1.03-1.22]	**0.01**	1.12	[1.03-1.22]	**0.01**
**Surgery within the first 15 days** (reference=no)	0.78	[0.37-1.68]	0.53	0.39	[0.12-1.21]	0.10	0.37	[0.13-1.07]	0.07
**Vegetation size** (per one mm increase)	1.05	[1.02-1.09]	**0.003**	1.05	[1.01-1.10]	**0.01**	1.06	[1.01-1.10]	**0.01**
**Perforation or regurgitation** (reference=no)	2.21	[1.00-4.87]	**0.05**	2.35	[0.92-5.96]	0.07	2.16	[0.92-5.11]	0.08
**Inoculum effect (InE)** (reference= absence of *blaZ* or InE)	2.32	[1.14-4.70]	**0.02**	2.47	[1.06-5.75]	**0.04**	2.38	[1.06-5.38]	**0.04**

NA: Not analyzed in the models because the variables comprising the Charlson comorbidity index were used instead of the total score
NS: Not selected during stepwise selection

© 2024 Jean B et al. *JAMA Network Open.*

eFigure 1. *blaZ* type frequency by clinical center

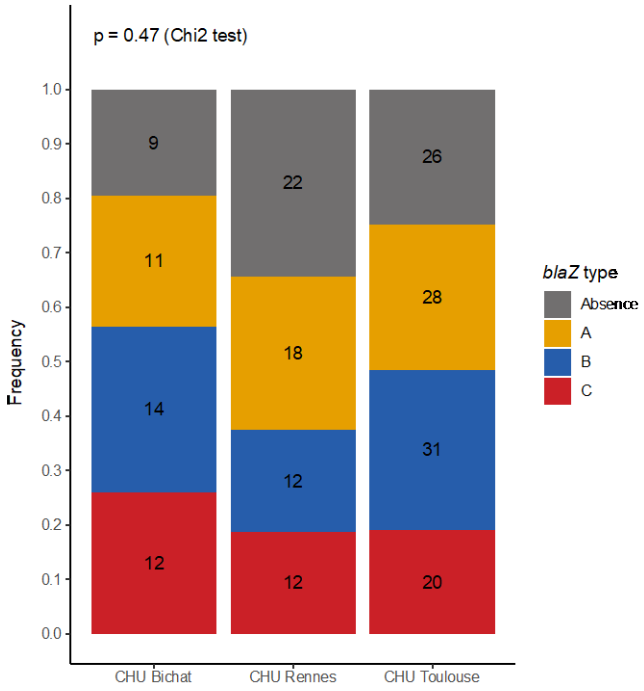

The β-lactamase type was determined on the basis of the amino acids at positions 128 and 216 encoded by the *blaZ* gene.

© 2024 Jean B et al. *JAMA Network Open.*

eFigure 2. One-month survival curves in MSSA left-sided infective endocarditis according to *blaZ* type

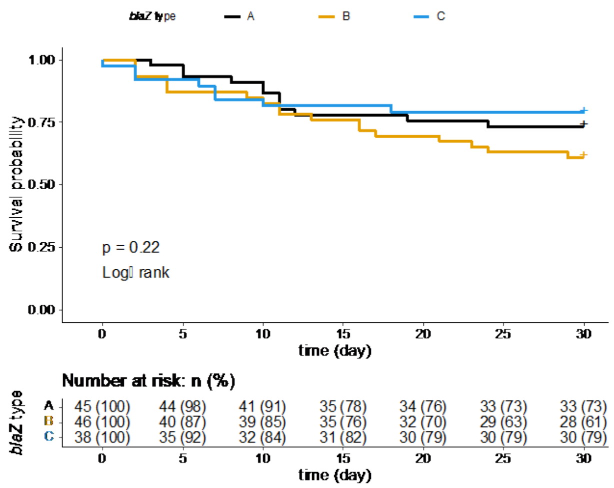

Survival curves in MSSA left-sided IE plotted using the Kaplan-Meier method and compared by *blaZ* type (in black: type A; in orange: type B; in blue: type C) using the log-rank test.

© 2024 Jean B et al. *JAMA Network Open.*

eFigure 3. One-month survival curves in MSSA left-sided infective endocarditis in patients infected with a *blaZ*-positive strain according to the presence or absence of an inoculum effect to the β-lactam received

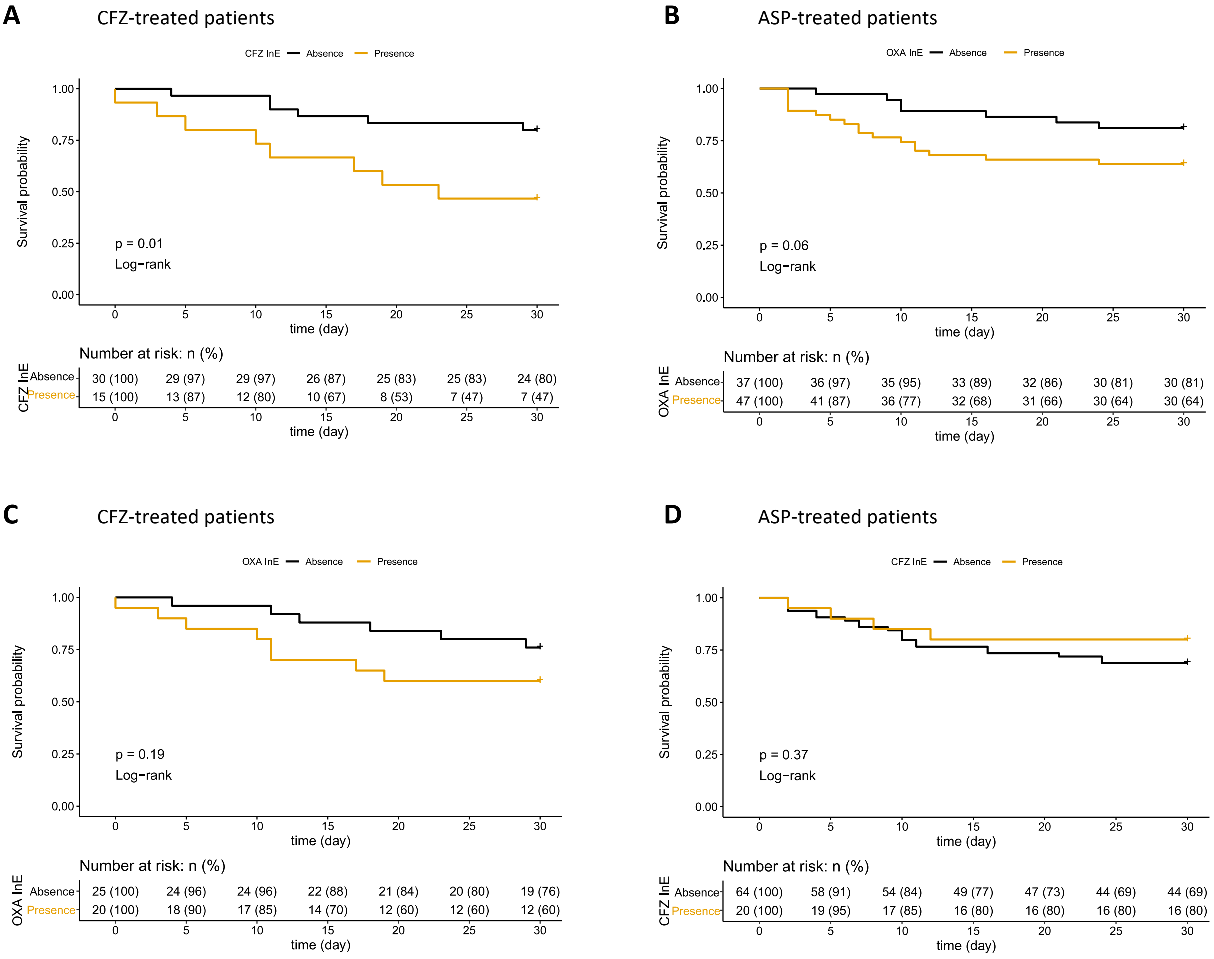

Survival curves in MSSA left-sided IE plotted using the Kaplan-Meier method in patients with a *blaZ*-positive strain in patients treated with CFZ and having a CFZ inoculum effect (**A**); in patients treated with ASP and having an OXA inoculum effect (**B**); in patients treated with CFZ and having an OXA inoculum effect (**C**); and in patients treated with ASP and having a CFZ inoculum effect (**D**). Comparisons were made using the log-rank test.

© 2024 Jean B et al. *JAMA Network Open.*
