# Supplementary material for: β-Lactam Inoculum Effect in Methicillin-Susceptible Staphylococcus aureus Infective Endocarditis
Source: JAMA Netw Open. 2024 Dec 20;7(12):e2451353. doi: 10.1001/jamanetworkopen.2024.51353 (PMC11662251; doi:10.1001/jamanetworkopen.2024.51353)
Supplement: Supplement 2. — Data Sharing Statement [file jamanetwopen-e2451353-s002.pdf]

Data Sharing Statement

Jean. β-lactam Inoculum Effect in Methicillin-Susceptible *Staphylococcus aureus* Infective Endocarditis. *JAMA Netw Open*. Published December 20, 2024.
doi:10.1001/jamanetworkopen.2024.51353

Data
Data available: Yes
Data types: Deidentified participant data
How to access data: via the corresponding author: delobel.p@chu-toulouse.fr
When available: With publication

Supporting Documents
Document types: None

Additional Information
Who can access the data: researchers whose proposed use of the data has been approved
Types of analyses: for any purpose
Mechanisms of data availability: after approval of a proposal
